# The ZNF750–RAC1 axis as potential prognostic factor for breast cancer

**DOI:** 10.1038/s41420-020-00371-2

**Published:** 2020-11-29

**Authors:** Alessio Butera, Matteo Cassandri, Francesco Rugolo, Massimiliano Agostini, Gerry Melino

**Affiliations:** 1 Department of Experimental Medicine, TOR, University of Rome “Tor Vergata”, 00133 Rome, Italy; 2 Present Address: Department of Oncohematology, Bambino Gesu’ Children’s Hospital, 00146 Rome, Italy

**Keywords:** Breast cancer, Tumour biomarkers

## Abstract

The human zinc finger (C2H2-type) protein ZNF750 is a transcription factor regulated by p63 that plays a critical role in epithelial tissues homoeostasis, as well as being involved in the pathogenesis of cancer. Indeed, missense mutations, truncation and genomic deletion have been found in oesophageal squamous cell carcinoma. In keeping, we showed that ZNF750 negatively regulates cell migration and invasion in breast cancer cells; in particular, ZNF750 binds and recruits KDM1A and HDAC1 on the *LAMB3* and *CTNNAL1* promoters. This interaction, in turn, represses the transcription of *LAMB3* and *CTNNAL1* genes, which are involved in cell migration and invasion. Given that ZNF750 is emerging as a crucial transcription factor that acts as tumour suppressor gene, here, we show that ZNF750 represses the expression of the small GTPase, Ras-related C3 botulinum toxin substrate 1 (RAC1) in breast cancer cell lines, by directly binding its promoter region. In keeping with ZNF750 controlling RAC1 expression, we found an inverse correlation between ZNF750 and RAC1 in human breast cancer datasets. More importantly, we found a significant upregulation of RAC1 in human breast cancer datasets and we identified a direct correlation between RAC1 expression and the survival rate of breast cancer patient. Overall, our findings provide a novel molecular mechanism by which ZNF750 acts as tumour suppressor gene. Hence, we report a potential clinical relevance of ZNF750/RAC1 axis in breast cancer.

## Introduction

ZNF750 is a zinc finger transcription factor that plays an important role in controlling the homoeostasis of epithelial tissue^1,2^. Indeed, ZNF750 deregulation results in neoplastic transformation^3^. ZNF750 consists of an atypical C2H2 zinc finger motif in the N-terminal domain, which is required for ZNF750 transcriptional function. In addition, two highly conserved PLNLS motifs, that are required for protein–protein interaction, are present in the C-terminal domain^4^. ZNF750 regulates epidermal differentiation by activating differentiation gene expression cooperatively with KLF4 and by inhibiting the expression of progenitor genes in association with KDM1A. In addition, ZNF750 has been described as a tumour suppressor protein in squamous cell carcinomas (SCCs) of the oesophagus and lung^5,6^. In SCCs, ZNF750 results frequently mutated, and truncation and missense mutations represent the most frequent genetic alterations. Notably, overexpression of ZNF750 inhibits cell proliferation and migration of SCC cells lines by regulating the expression of terminal differentiation-induced ncRNA (TINCR)^7^, through which it controls cancer cell proliferation and inhibits the expression of Laminin Subunit Gamma 2 (LAMC2), a component of Laminin-332 (ref. ^5^). Accordingly, low expression of ZNF750 has been associated with poor prognosis. We have recently shown that ZNF750 negatively regulates cell migration, invasion and proliferation in breast cancer cells. Specifically, ZNF750 binds and recruits KDM1A and HDAC1 on the Laminin Subunit Beta 3 (LAMB3) and Catenin Alpha Like 1 (CTNNAL1) promoters. This interaction in turn represses the transcription of LAMB3 and CTNNAL1 genes, two well-known proteins involved in cell migration and invasion^8^.

Breast cancer is the most common invasive cancer diagnosed in women. It is a complex disease that shows both inter- and intratumoral heterogeneity. Gene expression profiling studies have identified five distinct subtypes of breast cancers: luminal A, luminal B, HER2-enriched, basal-like and normal-like. Each of these subtypes is characterized by distinct disease progression, therapeutic response and clinical outcome. Therefore, a stratification of the patients is necessary to achieve better clinical outcome and for predicting the clinical course of the disease^9,10^. This is a general problem in clinical cancer research^11–13^, and current research is very active in this respect^14^, including for example on neuroblastoma^9,15–17^. To achieve this, novel molecular pathways that are implicated in the pathogenesis of cancer should be uncovered and identification of novel biomarkers is needed for predicting patient clinical outcome^15,16,18^.

Although in the last few years several studies have highlighted the role of ZNF750 as tumour suppressor gene, the molecular mechanisms underlying the inhibition of cancer onset and progression are not well characterized. In this study, we add a layer of complexity by showing that ZNF750 acts as tumour suppressor protein by transcriptional inhibition of Ras-related C3 botulinum toxin substrate 1 (RAC1), a member of the Rho/Rac GTPase family^19^.

## Results

### RAC1 is upregulated in breast cancer cells and its expression is controlled by ZNF750

**Fig. 1:**
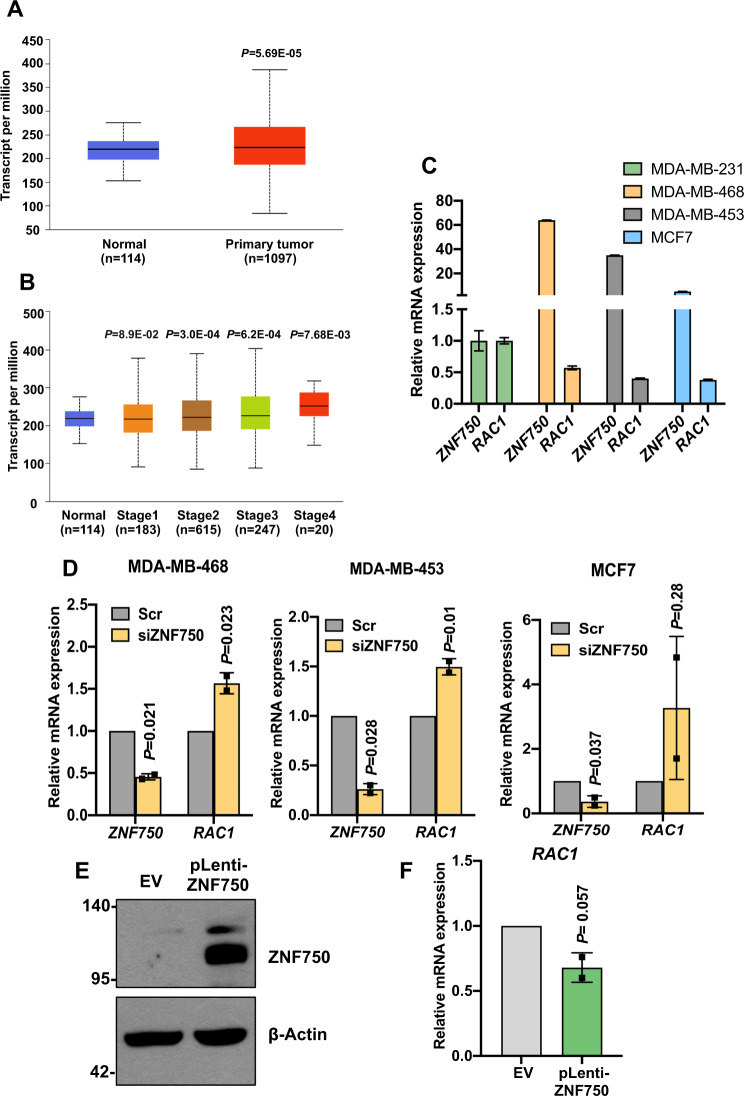
RAC1 expression is controlled by ZNF750 in breast cancer cells. **A**, **B** Bioinformatic analysis showing the increased expression of RAC1 in breast cancer and in the different cancer stages. The graphs were directly downloaded from the UALCAN website (http://ualcan.path.uab.edu/index.html). The datasets analysed were from TCGA. **C** Expression of ZNF750 and RAC1 across several breast cancer cell lines. Data represent the mean of three technical replicates (*N* = 3, PCR runs) ± SD and are representative of one experiment. **D** Depletion of ZNF750 caused RAC1 upregulation at the mRNA level. Bars represent the mean ± SD of two independent experiments. **E** Immunoblot showing the overexpression of ZNF750 in transduced MDA-MB-231. **F** Overexpression of ZNF750 represses RAC1 mRNA expression. Bars represent the mean ± SD of two independent experiments (*N* = 2).

We have previously demonstrated that ZNF750 acts as a tumour suppressor in breast cancer^8^. In particular, ZNF750 inhibits migration and invasion of the breast cancer cell lines by downregulating Wnt signalling. Among the several players involved in the Wnt signalling, RAC1 mediates the transduction of the non-canonical Wnt pathway and therefore is implicated in the establishment of cell polarity and cell migration^20^. We have recently found that *RAC1* was upregulated after ZNF750 depletion in breast cancer cell lines. To gain insight into the role of RAC1 in breast cancer, we first performed a comprehensive analysis of the expression of RAC1 in breast cancer tissue samples. As shown in Fig. 1A, high levels of RAC1 mRNA are found in primary tumour when compared with the normal tissue. Moreover, RAC1 expression positively correlates with tumour stage (Fig. 1B). To further explore the relationship between ZNF750 and RAC1, we tested RAC1 expression by qPCR in several breast cancer cell lines characterized by different ZNF750 expression levels: MDA-MB-468 (high expression), MDA-MB-453 (high expression), MCF7 (moderate expression) and MDA-MB 231 (low expression). Interestingly, we observed that RAC1 expression inversely correlated with ZNF750. In particular, as shown in Fig. 1C, in the high-ZNF750-expressing MDA-MB-468 and MDA-MB-453 cell lines, RAC1 was expressed at lower levels as compared to the more aggressive MDA-MB-231. Conversely, MDA-MB-231 cell line, which was previously characterized by the lowest ZNF750 levels^8^, showed the highest levels of RAC1 (Fig. 1C). In order to confirm that the RAC1 expression was ZNF750-dependent, we depleted ZNF750 using a specific siRNA in MDA-MB468, MDA-MB-453 and MCF7. Interestingly, ZNF750 depletion resulted in a significant RAC1 upregulation in all the three breast cancer lines (Fig. 1D). On the contrary, overexpression of ZNF750 in MDA-MB-231 (Fig. 1E) cells resulted in a reduction of RAC1 mRNA levels (Fig. 1F). Collectively, these data suggest that ZNF750 controls the expression of RAC1 in breast cancer cell lines.

### ZNF750 directly binds RAC1 promoter region

**Fig. 2:**
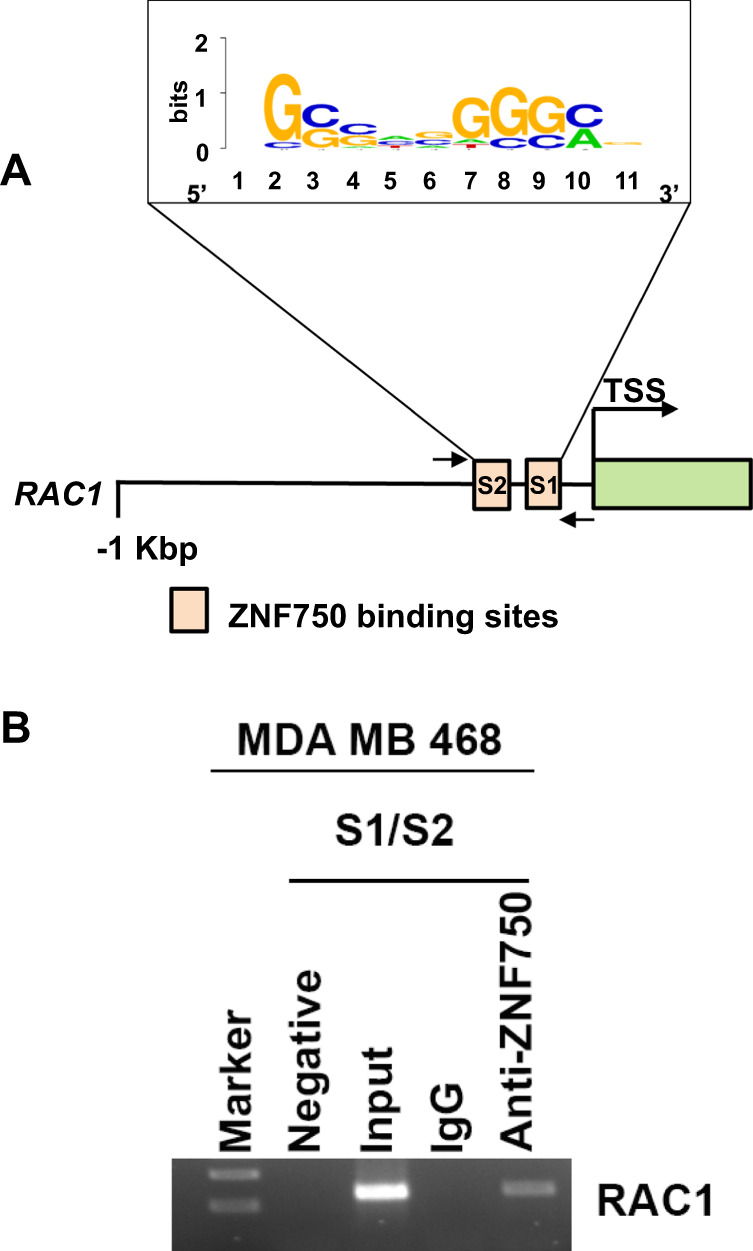
ZNF750 binds the promoter region of RAC1. **A** Schematic representation of the scanned RAC1 promoter region with two putative binding sites for ZNF750 containing the consensus sequence reported. **B** Chromatin immunoprecipitation assay showing the binding of ZNF750 to *RAC1* promoter region. Arrows indicate the primer position for PCR amplification.

To gain insight into the molecular mechanism by which ZNF750 regulates the expression of RAC1, we performed a careful analysis of RAC1 proximal promoter using the JASPAR^21^ and MEME suite websites^22^. By scanning the 1 kbp-upstream region of *RAC1* gene, we found two putative binding sites for ZNF750 located at 882 bp and 897 bp, respectively (Fig. 2A). To investigate whether ZNF750 directly binds the promoter region of RAC1, we performed chromatin immunoprecipitation (ChIP) in MDA-MB-468 cells. As shown in Fig. 2B, endogenous ZNF750 directly binds the RAC1 promoter region suggesting a potential direct transcriptional control. Overall these data indicate that ZNF750 negatively modulates RAC1 expression through a direct binding to its promoter.

### ZNF750 and RAC1 negatively correlate in human breast cancer

**Fig. 3:**
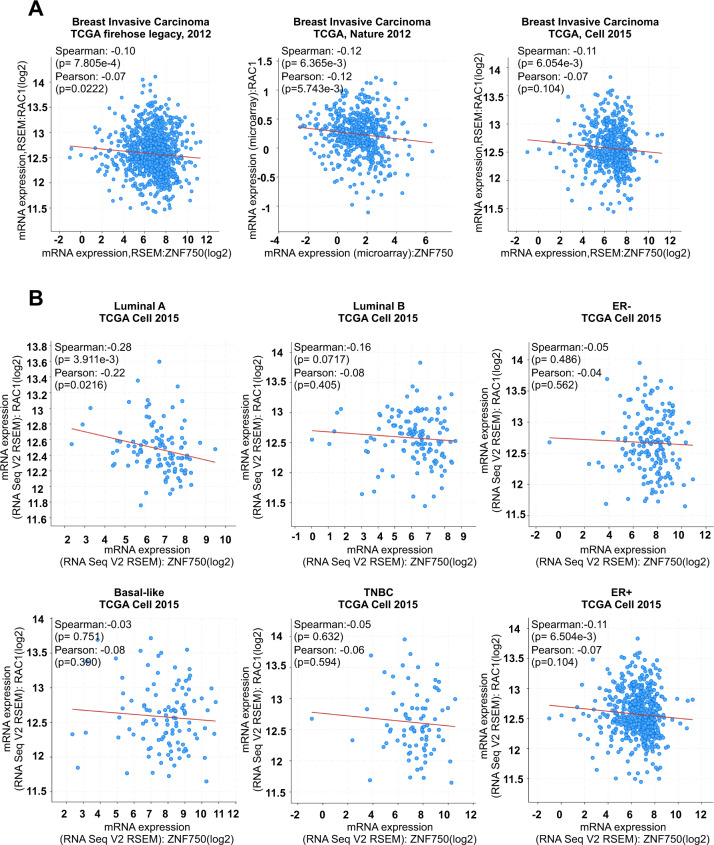
ZNF750 and RAC1 expressions negatively correlate in breast cancer patients. **A** Bioinformatic analysis showing a negative correlation between ZNF750 and RAC1 in breast invasive carcinoma. The datasets analysed were TCGA, Cell 2015; TCGA Nature 2012; TCGA Firehose Legacy, 2012. **B** ZNF750 and RAC1 inversely correlated in different breast cancer subtypes. The dataset analysed was TCGA, Cell 2015. For **A** and **B**, the analysis was carried out using the cBioPortal for Cancer Genomics database (http://www.cbioportal.org).

**Fig. 4:**
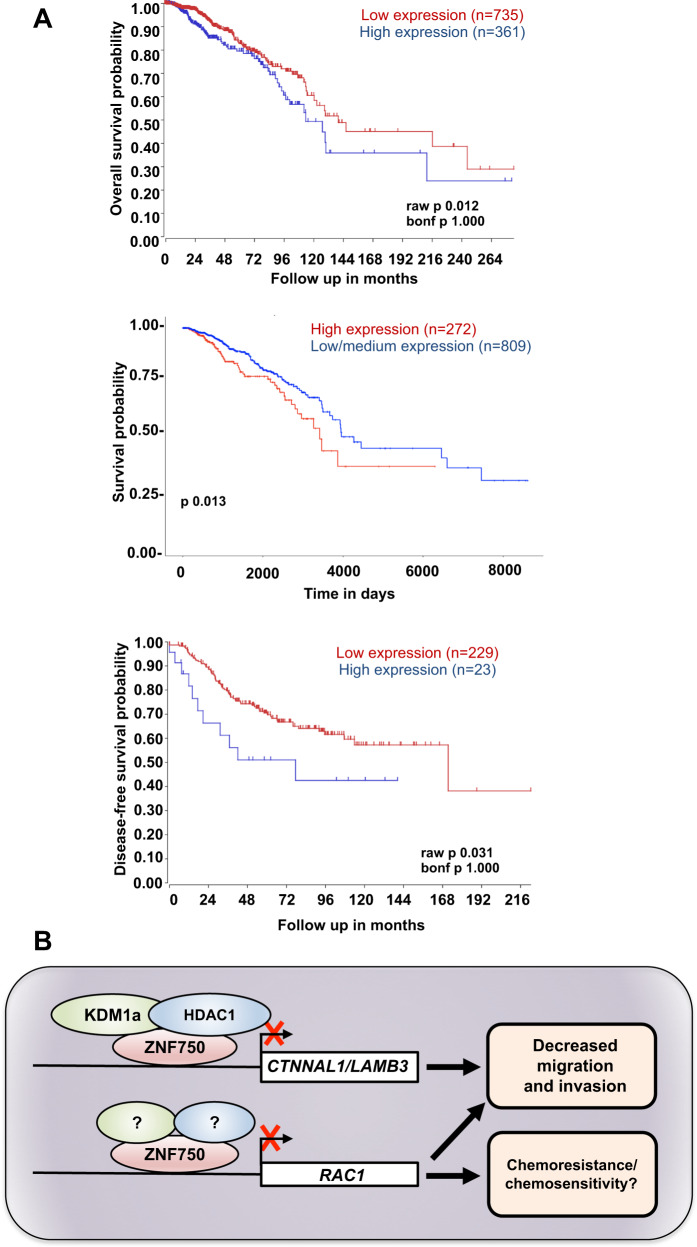
High level of RAC1 is associated with reduced patient survival. **A** Kaplan–Meier survival analysis showing that higher expression of RAC1 associates with a poor patient survival. The analysis was performed across several datasets of breast invasive carcinoma using cBioPortal for Cancer Genomics database, the R2 Genomic Platform (https://r2.amc.nl) and UALCAN website (http://ualcan.path.uab.edu/index.html). **B** Schematic representation of ZNF750 role in breast cancer. ZNF750 represses breast cancer migration and invasion by recruiting the epigenetic modifiers KDM1a and HDAC1 on *CTNNAL1* and *LAMB3* promoters (ref. ^8^). Furthermore, ZNF750 represses *RAC1* expression by binding to its promoter, therefore modulating the cytoskeleton remodelling and possibly affecting migration and chemoresistance in breast cancer.

RAC1 expression increases with the tumour progression, and hyperactivation and/or overexpression are associated with a very poor prognosis^23^, while, on the contrary, ZNF750 levels significantly decrease with the breast cancer stage and negatively correlate with cancer aggressiveness. Then, we asked whether the negative correlation between ZNF750 and RAC1 may have a clinical relevance. Thus, using the cBioPortal database^24,25^, we asked whether the negative correlation between ZNF750 and RAC1 observed in breast cancer cell lines was also conserved in human breast cancer by using publicly available breast cancer datasets. As shown in Fig. 3A, we found a significant negative correlation between ZNF750 and RAC1 in all the breast invasive carcinoma datasets analysed. Given that breast cancer is characterized by high molecular and cellular heterogeneity^26^, we analysed the correlation between ZNF750 and RAC1 among the different breast cancer subtypes. The bioinformatic analysis reported a significant correlation (*p* < 0.05) only for the luminal A subtype (Fig. 3B). Remarkably, RAC1 expression was discriminatory of good and bad prognosis in breast cancer patients. Indeed, as shown in Fig. 4, high levels of RAC1 were associated with a worse survival in all the cancer datasets assessed. Overall our data indicate that ZNF750 acts as tumour suppressor gene in breast cancer by negatively regulating the expression of RAC1.

## Discussion

In the last decade, OMICS technologies have contributed to our understanding of the pathogenesis of cancer^27–30^ leading to the development of precision oncology^31,32^, where selection of the patients for treatment is fundamental for better therapy outcome^33^. Therefore, identification of novel biomarkers for guiding treatment selection is a key requirement^14,34–36^.

We have previously identified the involvement of p63 in the progression of breast cancer through regulation of Sonic Hedgehog signalling^37^ in epithelial stem cells^38^ that is able to interact with p53^39^ or IKK^40^ to keep epithelial homoeostasis^41,42^ or to kill^43^. Although p63 belongs to the p53 family^44–46^, this is a unique property of p63^47^.

In the current study, we show that the axis ZNF750/RAC1 may function as a potential biomarker for predicting patient survival in breast cancer. In particular, ZNF750 transcriptionally represses the expression of RAC1, a member of the Rho/Rac GTPase family^48^ by binding its promoter region. In agreement, ZNF750 expression negatively correlates with RAC1 expression in human breast cancer and in particular in luminal A subtypes. RAC1 is a molecular switch that can be found either in an active state, when complexed to GTP, and in an inactive state when bound to GDP. When activated, RAC1 is implied in the cell migration, through a cytoskeleton rearrangement, and in cell survival. In the latter case, a large number of studies have demonstrated that the cell survival potential occurs via activation of the MAPK kinase pathway, through the formation of the Raf/MEK/ERK complex^49^. In addition, RAC1 directly interacts with PI3K, stimulating the PI3K/AKT signalling and survival^50–52^. Of relevance, a somatic mutation of RAC1 has been found as an oncogenic driver in melanoma, head and neck and prostate cancers^53,54^. Moreover, high expression of RAC1 was shown to be associated with poor outcome in several human cancers, such as colorectal cancers, and leukaemia^19,55,56^. In agreement with this scenario, our findings provide evidences for a parallel and/or alternative molecular mechanism by which ZNF750 functions as tumour repressor in breast cancer. In fact, we would like to speculate that besides repressing LAMB3 and CTNNAL1 expression, ZNF750 might also inhibit migration and invasion in breast cancer by repressing the expression of RAC1 (Fig. 4B). Despite its role in migration and survival, ZNF750 is also involved in resistance to chemoradiotherapy. Indeed, high levels of ZNF750 are associated with a good response to chemoradiotherapy in oesophageal SCC^57^. Interestingly, recently it has been shown that overexpression of RAC1 confers resistance to neoadjuvant chemotherapy in triple negative breast cancer^23,58^. Therefore, it is tempting to hypothesize that ZNF750 sensitizes cancer cells to chemotherapy by repressing the expression of RAC1. Although this should be tested in clinical setting, our data may also suggest that the ZNF750/RAC1 axis may serve as a novel candidate biomarker for chemoradiotherapy sensitivity.

In cancer, treatment is becoming crucial to identify biomarkers that will allow the stratification of the patients in order to select the most appropriate treatment for a subgroup of patients. Here, we identify the axis ZNF750/RAC1 as a potential novel prognostic biomarker for predicting clinical outcome in breast cancer. Moreover, our findings highlight a possible alternative molecular mechanism by which ZNF750 functions as tumour suppressor gene in breast cancer.

## Methods

### Cell culture and transfection

All cell lines used were obtained from American Type Culture Collection (ATCC) and maintained at 37 °C in 5% CO_2_ in culture medium. MCF7 (adenocarcinoma), MDA-MB-468 (ductal carcinoma, basal like-1), MDA-MB-453 (carcinoma, LAR) and MDAMB-231 (adenocarcinoma, mesenchymal-stem-like) were grown in Dulbecco’s Modified Eagle’s medium with 4.5 g/l glucose supplemented with 250 μM l-glutamine (Gibco), penicillin/streptomycin 1 U/ml (Gibco) and 10% FBS (Invitrogen). MDA-MB-231 stably overexpressing ZNF750 were previously generated and used for the study. Silencing was performed using 50 nM of ZNF750-specific siRNAs with Lipofectamine RNAimax according to the manufacturer’s protocol. Forty-eight hours after transfection, cells were harvested for experimental procedures.

### RNA isolation and quantitative real-time PCR

Total RNA from cells was isolated using an RNeasy minikit (Qiagen) according to the manufacturer’s instructions. RNA samples were treated with RNase-free DNase I (Qiagen), and RNA was quantified using a NanoDrop spectrophotometer (Thermo Scientific). Total RNA was reverse transcribed using the SensiFAST cDNA Synthesis kit (Bioline) according to the manufacturer’s protocol. qRT-PCR was performed using GoTaq qPCR Mastermix (Promega) with SYBR Green ready mix. The expression of each gene was defined according to the threshold cycle (Ct), and relative expression levels were calculated by using the 2−ΔΔCt method after normalization to the expression of the housekeeping gene β-actin. All the primer sequences are listed as follows: ZNF750 for: 5′-AGCTCGCCTGAGTGTGAC-3′; ZNF750 rev: 5′-TGCAGACTCTGGCCTGTA-3′; RAC1 for: 5′-GCTGACTCCCATCACCTATCC-3′; RAC1 rev: 5′-CGAGGGGCTGAGACATTTACAA-3′; β-ACTIN for: 5′-GTTGCTATCCAGGCTGTG-3′; β-ACTIN rev: 5′-AATGTCACGCACGATTTCCCG-3′.

### Western blot analysis

For immunoblot analysis, proteins were extracted with RIPA buffer containing cocktail inhibitors (Roche), separated on SDS polyacrylamide gels and then transferred onto nitrocellulose membranes (GE Healthcare) by a wet-transfer system. Membranes were blocked with TBS-0.1% Tween and 10% milk and incubated overnight with primary antibodies. The following day, membranes were washed and then incubated with the appropriate horseradish peroxidase-conjugated secondary antibody. Proteins were visualized with the Super Signal chemiluminescence kit (Thermo Scientific). The following antibodies were used: anti-ZNF750 (1:1000; Sigma HPA023012) and anti-β-actin (1:50,000; Sigma A5441).

### ChIP assay

MDA-MB-468 were cross-linked for 10 min in a solution containing 1% formaldehyde. After fixation, ChIP assay was performed using a MAGnify ChIP system (Invitrogen) according to the manufacturer’s instructions. In brief, cells were lysed and then sonicated to obtain chromatin fragments of ~250–400 bp. The lysate was immunoprecipitated using an anti-ZNF750 specific antibody (Sigma HPA023012), and nonspecific IgG as a negative control. Collected DNA fragments were tested by canonical PCR. In order to confirm the specificity of the antibody, a negative control region belonging to the β-actin promoter was chosen. The primer sequences used for the amplification reaction are reported as follows: RAC1 promoter for: 5′- TCCGAGCATTCCCGAAGTCC-3′; RAC1 promoter rev: 5′-AAATGGCCGCTCCACTCAC-3′.

### Bioinformatics analysis

For bioinformatic analysis, the following websites were used: cBioPortal for Cancer Genomics, http://www.cbioportal.org^24,25^, UALCAN, http://ualcan.path.uab.edu^59^ and R2 (R2: Genomics Analysis and Visualization Platform (http://r2.amc.nl)).

The UALCAN website was interrogated to analyse the expression levels of RAC1 between breast cancer and the normal tissue counterpart. Expression levels were obtained by RNA sequencing of normal and tumoral tissues and results were represented as transcripts per million (TPM). Even more, the UALCAN website reported the Kaplan–Meier survival curve of patients stratified with high (with TPM values above upper quartile) and low (with TPM values below upper quartile) RAC1 levels. The cBioPortal was interrogated to analyse the ZNF750–RAC1 correlation in TCGA Breast Cancer Invasive Carcinoma Datasets. The following datasets were analysed: TCGA, firehose legacy, 2012; TCGA Nature 2012 (ref. ^60^); TCGA, Cell 2015 (ref. ^61^). The latter was further used to study the correlation among the different breast cancer subtypes. The R2 platform was interrogated to enlarge the number of datasets for the Kaplan–Meier analysis. The interrogated datasets were: Breast Tumour GSE21653 (ref. ^62^) and Tumour Breast Invasive Carcinoma-TCGA.

### Statistical analysis

All results are expressed as the mean ± SDs. *p*-Values < 0.05 were considered statistically significant.
